# Mapless Path Planning for Mobile Robot Based on Improved Deep Deterministic Policy Gradient Algorithm

**DOI:** 10.3390/s24175667

**Published:** 2024-08-30

**Authors:** Shuzhen Zhang, Wei Tang, Panpan Li, Fusheng Zha

**Affiliations:** 1 School of Mechanical and Electrical Engineering, Lanzhou University of Technology, Lanzhou 730000, China; weit38344@gmail.com (W.T.); 17339905809@163.com (P.L.); 2 State Key Laboratory of Robotics and System (HIT), Harbin Institute of Technology, Harbin 150001, China; zhafusheng@hit.edu.cn

**Keywords:** mobile robot, mapless path planning, deep deterministic policy gradient, multi-step update, artificial potential field

## Abstract

In the traditional Deep Deterministic Policy Gradient (DDPG) algorithm, path planning for mobile robots in mapless environments still encounters challenges regarding learning efficiency and navigation performance, particularly adaptability and robustness to static and dynamic obstacles. To address these issues, in this study, an improved algorithm frame was proposed that designs the state and action spaces, and introduces a multi-step update strategy and a dual-noise mechanism to improve the reward function. These improvements significantly enhance the algorithm’s learning efficiency and navigation performance, rendering it more adaptable and robust in complex mapless environments. Compared to the traditional DDPG algorithm, the improved algorithm shows a 20% increase in the stability of the navigation success rate with static obstacles along with a 25% reduction in pathfinding steps for smoother paths. In environments with dynamic obstacles, there is a remarkable 45% improvement in success rate. Real-world mobile robot tests further validated the feasibility and effectiveness of the algorithm in true mapless environments.

## 1. Introduction

Currently, mobile robot technology is widely utilized in various domains such as industrial automation, smart homes, and autonomous driving, playing a pivotal role in improving production efficiency, enhancing service quality, and exploring future lifestyles [1]. To ensure stable operation in diverse environments, efficient and safe path planning strategies are essential for mobile robots [2]. Currently, path planning algorithms can be divided into traditional methods and intelligent methods [3,4]. Traditional path planning methods can further be categorized into global and local approaches. The former includes algorithms such as the Dijkstra algorithm [5], the A* algorithm [6], the RRT algorithm [7], and genetic algorithms [8], which rely on complete prior information about the environment. The latter encompasses methods like the Dynamic Window Approach (DWA) [9] and artificial potential field (APF) [10], which improved real-time performance but were prone to local optimal solutions. Nevertheless, conventional path planning methods often suffer from reliance on maps and inadequate real-time performance. As the working environment of the mobile robots becomes increasingly intricate, traditional path planning methods are no longer able to fulfill the practical task requirements [11]. In recent years, reinforcement learning (RL) has demonstrated significant potential in path planning [12]. Through continuous interaction with the environment, RL is capable of learning optimal action strategies. For example, algorithms like Q-learning [13] and SARSA [14] can identify optimal paths in mapless environments through trial and error. The Q-learning algorithm learns optimal paths by updating the Q-value table. However, in complex environments, the excessive number of state–action pairs increases memory consumption and dimensionality issues, thereby limiting its effectiveness in real-time applications [15,16]. Deep reinforcement learning (DRL) [17] utilizes deep neural networks to extract environmental features, effectively mapping high-dimensional perceptual information to low-dimensional state spaces. This approach successfully addressed the dimensionality problem, significantly improving path planning efficiency and accuracy. In 2013, the Deep Q-Network (DQN) algorithm, as the DeepMind team proposed, was demonstrated to be able to learn control strategies directly from raw pixel inputs [18]. This revolutionary end-to-end algorithm from perception to action addressed the dimensionality problem in Q-learning [19]. However, its applicability was primarily limited to discrete action spaces and it encountered challenges when adapting to real-world multi-task continuous action spaces. To tackle this issue, the DeepMind team introduced the Deep Deterministic Policy Gradient (DDPG) algorithm [20], which has demonstrated efficient learning in continuous action spaces. Subsequent advancements by both academia and industry have further refined this algorithm; for instance, Dong et al. enhanced exploration efficiency and algorithm convergence speed through the incorporation of prior knowledge and adoption of the ε-greedy algorithm for adaptive exploration. However, the efficacy of their work was solely evaluated in a predetermined simplified grid environment, and the algorithm’s performance was heavily reliant on precise parameter configurations [21]. Han Zhang et al. enhanced network output action strategies through the utilization of the artificial potential field method for intervention, resulting in improved path smoothness and shortened path length. Nonetheless, their convergence speed was suboptimal, and experimental validation relied on simulated environments [22]. Li et al. introduced dual actor and critic networks alongside an optimal action selection mechanism, leading to enhanced stability and superior performance. However, the intricacy of this structure resulted in increased computational demands and more complex parameter adjustment processes [23].

Despite the advancements in prior knowledge, adaptive exploration methods, and network structure optimization, these improvements are often confined to simplified test environments and rely on precise parameter settings, typically leading to slow convergence, which limits their generalizability and efficiency in practical applications. To address these issues, we have applied several improvements to the DDPG algorithm, including the following: (1) Designing continuous state and action spaces to enhance the mobile robot’s response speed and safety in complex environments, thereby addressing the inadequacies of traditional state spaces in real-time decision-making. (2) Implementing a multi-step update mechanism to comprehensively consider future rewards, improving the stability and convergence speed of policy optimization and addressing the slow convergence problem of traditional single-step updates. (3) Integrating the artificial potential field method to design a continuous reward function, guiding the mobile robot quickly and effectively toward the target direction. (4) Designing a dual-noise exploration mechanism to introduce higher randomness during the mobile robot’s learning process, avoiding local optima and improving exploration efficiency. These enhancements were aimed at achieving efficient, stable, and high-quality path planning for mobile robots operating in mapless environments.

## 2. DDPG Algorithm

**Figure 1:**
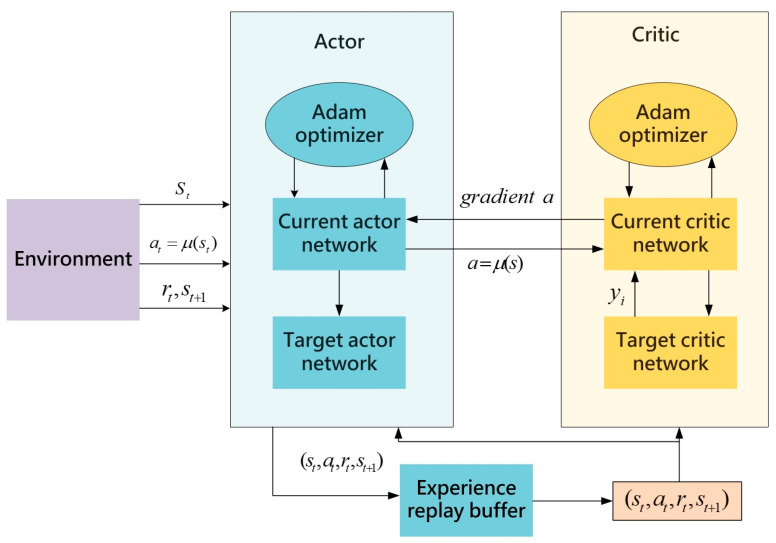
The architecture diagram of the DDPG algorithm.

The Deep Deterministic Policy Gradient (DDPG) algorithm [20] combines the advantages of the Deep Q-Network (DQN) [18] and the actor–critic structure to achieve efficient learning and decision-making in continuous action spaces. The DDPG algorithm utilizes an actor–critic framework, in which the actor network takes the current state as input and outputs a deterministic action. This deterministic action generation allows for precise decision-making in continuous action spaces, while the critic network evaluates these actions by inputting state–action pairs and outputting corresponding Q-values. The dual-network structure effectively facilitates policy generation and value evaluation, enabling DDPG to excel in complex continuous action spaces. To enhance training stability, the DDPG algorithm introduces a replay buffer to store interaction experiences with the environment. During training, random sampling from the replay buffer breaks correlation between samples, reduces the impact of non-stationary data on the training process, and improves overall training stability. In addition, DDPG incorporates target networks, which are delayed-update copies of the actor and critic networks. The target networks aim to mitigate rapid changes in value estimates that may destabilize the training process. The slower parameter updates of the target networks provide a more stable learning target, further enhancing the stability of the training process. During training, the agent continuously interacts with the environment, accumulating experiences and storing them in the replay buffer. The algorithm periodically samples experiences from the buffer to train and update the parameters of the actor and critic networks. Serving as delayed-update copies of these networks, the target networks offer a relatively stable learning target, effectively preventing rapid changes in value estimates and improving overall stability and efficiency during training. The architectural diagram of this algorithm is depicted in Figure 1:

### 2.1. DDPG Algorithm Flow

#### 2.1.1. Initialize Networks

The DDPG algorithm begins by initializing the current policy network $\mu \left(s\right|\theta^{\mu })$ and the current value network $Q(s,\;a|\theta^{Q})$. Subsequently, the target policy network $\mu \prime \left(s\right|\theta^{\mu \prime })$ and the target value network $Q\prime \left(s,\;a\right|\theta^{Q\prime })$ are initialized with their weights $\theta^{\mu \prime }$ and $\theta^{Q\prime }$ set to match those of the main networks in order to facilitate a smooth transition during the initial learning stages. An experience playback buffer is then created and initialized for storing interaction data between the intelligent agent and its environment, encompassing states, actions, rewards, and subsequent states. It enables random sampling of small batches of experiences for training throughout the process, effectively breaking temporal correlations between successive experiences and mitigating non-smooth data’s impact on training stability.

#### 2.1.2. Interaction and Storage

In the current policy network, the input is represented by the state S, and the output is a deterministic action $a=\mu (s|\theta^{\mu })$. The relationship between state S and action a can be described by the state transition function $P(S_{t+1}|S_{t},a_{t})$, which represents the probability of transitioning to the next state $S_{t+1}$ given the current state $S_{t}$ and action $a_{t}$. Here, μ denotes the policy function parameterized by $\theta^{\mu }$. In the current value network, the input consists of both state and action, with the corresponding Q-value $Q=Q(S,\;a|\theta^{Q})$ as output. The Q-value signifies the expected return from taking action under the current policy parameterized by $\theta^{Q}$. At each time step t, an action $a_{t}$ is selected based on current state $S_{t}$ using the policy network to generate actions while incorporating exploration noise $N_{t}$ to encourage exploration. It can be expressed as $a_{t}=\mu \left(S_{t}|\theta^{\mu }\right)+N_{t}$. Following the execution of action $a_{t}$, interaction with the environment results in the acquisition of new state $S_{t+1}$ and corresponding reward $r_{t}$. The agent then stores the experience data $\left(S_{t},a_{t},r_{t},S_{t+1},\mathrm{done}\right)$ into an experience replay buffer. Here, the variable “done” is a boolean flag that indicates whether the agent has reached a terminal state. If the agent has successfully completed the task or entered a failure state, the value of “done” is set to True; otherwise, it is set to False. The pseudocode is shown in Table 1.

#### 2.1.3. Sampling and Training

Randomly sample a minibatch $\left(S_{t},\;a_{t},\;r_{t},\;S_{t+1},\;\mathrm{done}\right)$ from the experience replay buffer and calculate the target Q-value using the target policy network and target value network:

(1) $$y_{i}=r_{i}+\lambda \;Q\prime (S_{i+1},\mu \prime (S_{i+1}|\theta^{\mu \prime })|\theta^{Q\prime })$$

where λ is the discount factor, reflecting the importance of future rewards.

Calculate the loss of the critic network:

(2) $$L=\frac{1}{N}\sum_{i=1}^{N}{(y_{i}\;-\;Q(S_{i},a_{i}|\theta^{Q}))}^{2}$$

The critic network aims to minimize the loss function L by reducing the mean squared error (MSE) between the predicted Q-value $Q(S_{i},a_{i}|\theta^{Q})$ and the target value $y_{i}$. This minimization is achieved using gradient descent.

The actor update formula is

(3) $$\nabla_{\theta^{\mu }}J\approx \frac{1}{N}\sum_{i}\nabla_{a}Q\left(S,a|\theta^{Q}\right){|}_{S=S_{i},a=\mu \left(S_{i}\right)}\nabla_{\mu }\mu \left(S\right|\theta^{\mu }){|}_{S_{i}}$$

The actor network is updated by maximizing the Q-value estimated by the critic network for the actions it selects. This maximization is performed using gradient ascent.

The target networks are updated using a soft update method to enhance stability during the learning process:

(4) $$\theta^{Q\prime }\leftarrow \tau \theta^{Q}+\left(1-\tau \right)\theta^{Q\prime }$$

(5) $$\theta^{\mu \prime }\leftarrow \tau \theta^{\mu }+(1-\tau {)\theta }^{\mu \prime }$$

where τ represents the update coefficient. Through these steps, the DDPG algorithm continuously iterates and improves policy performance, ultimately achieving efficient mastery of complex continuous action tasks.

## 3. Improved DDPG Algorithm

### 3.1. Designing Continuous State and Action Spaces

In the environmental simulation for the mobile robot, the construction of the state space necessitates a comprehensive consideration of LiDAR data, action history, and the relative position and distance between the mobile robot and the target point. The incorporation of rich state information not only furnishes a comprehensive environmental background for decision-making but also serves to optimize navigation efficiency, thereby enhancing the robot’s response speed and safety in complex environments. The state vector $S_{t}$ is defined as $S_{t}=[L_{t},\;d_{t},\;\theta_{t},\;v_{t-1},\;w_{t-1}]$, where $L_{t}=[l_{1},\;l_{2},\;l_{3},\;l_{4}\ldots l_{20}]$ represents a 20-dimensional vector denoting distances to obstacles detected by LiDAR in 20 different directions. By utilizing a 20-dimensional vector, the mobile robot is able to ensure environmental perception accuracy while also considering computational efficiency. This enables the robot to accurately capture details of the surrounding environment and make real-time decisions efficiently. The variable $d_{t}$ represents the distance from the robot’s current position to the target point, aiding in determining direction and speed of movement. $\theta_{t}$ represents the yaw angle difference from the robot’s current orientation to the target point within a range of (−π,π). Negative values indicate that the destination is on the left side of the moving robot, while positive values indicate that it is on its right side. The yaw angle difference assists in adjusting direction towards reaching the target point. In this study, $v_{t-1}$ denotes the linear velocity of the robot at time t − 1, ranging from (0, 1). This offers insights into the robot’s previous movements, aiding in forecasting its future motion state and facilitating corresponding adjustments. $w_{t-1}$ represents the angular velocity of the robot at time t − 1, with a range of (−1,1), which enables the robot to make adaptable turning decisions in intricate environments for obstacle avoidance and path maintenance; negative velocity indicates leftward turns while positive indicates rightward turns.

### 3.2. Multi-Step Update

Traditional single-step methods update the value estimates of state–action pairs based on the immediate rewards at each time step and therefore lead to a slow learning process in the presence of sparse or delayed reward signals. This is due to the reliance of single-step updating solely on current immediate rewards, without consideration for future rewards, thereby impeding efficient strategy acquisition in reward-sparse environments. Furthermore, susceptibility to non-stationary data interference in complex tasks renders single-step updating methods unstable and hinders convergence speed during the learning process. This study used a multi-step updating method to avoid the limitations of single-step updates. By considering not only immediate rewards but also future rewards, multi-step updating enables intelligent agents to take into account long-term effects in the decision-making process. This expanded perspective allows intelligent agents to identify actions that may not yield high immediate rewards in complex environments, but are more advantageous in the long run. The approach is able to offer more comprehensive feedback, which assists intelligent agents in accurately evaluating long-term payoffs and facilitates the swift identification and adoption of strategies. Consequently, multi-step updating serves to diminish uncertainty and variance during the learning process, thereby enhancing the stability and efficacy of intelligent strategy optimization. According to references [24,25,26], experimental results indicated that multi-step updates not only improved the convergence speed of the algorithm but also significantly enhanced real-time performance. The representation of the multi-step update is as follows:

(6) $$y_{i}=\sum_{k=0}^{n-1}\lambda^{k}r_{i+k}+\lambda^{n}Q\prime (S_{i+n},\mu \prime (S_{i+n}|\theta^{\mu^{\prime }})|\theta^{Q\prime })$$

where λ is the discount factor, and $r_{i+k}$ is the immediate reward at time step i + k. $Q\prime (S_{i+n},\mu \prime (S_{i+n}|\theta^{\mu^{\prime }})|\theta^{Q\prime })$ represents the target Q-value at time step t+N computed by the value target network.

Calculate the loss of the critic network:

(7) $$L=\frac{1}{N}\sum_{i=1}^{N}{(y_{i}-Q(S_{i},a_{i}|\theta^{Q}))}^{2}$$

where $Q(S_{i},a_{i}|\theta^{Q})$ is the estimated value from the current value network. Use the gradient descent method to minimize the loss function L and update the parameters $\theta^{Q}$ of the critic network. With multi-step updating, the algorithm can learn the optimal policy faster, improving the efficiency and stability of learning. Additionally, this method can better handle reward delay issues in complex environments, making reinforcement learning algorithms more effective in practical applications.

### 3.3. Designing the Reward Function

The formulation of the reward function has a direct impact on the overall performance and task success rate in mobile robot path planning and navigation strategies. Drawing inspiration from the artificial potential field method [10], which is applied widely in adaptive path adjustment, obstacle avoidance, and target tracking for mobile robots, this study introduces an innovative reward function to comprehensively enhance the navigation capability of mobile robots in complex environments. The reward function of the design takes multiple key factors into account, not only focusing on the task completion of the mobile robot to finally reach the target, but also optimizing the path selection and behavioral adjustment of the intelligent body through the fine reward mechanism of each step. The designed reward function includes the following aspects.

#### 3.3.1. Target Reward and Penalty

Target Reward: Upon successful arrival at the designated destination, the system will administer a substantial positive reinforcement to underscore the paramount significance of task accomplishment. Based on experience, the reward for reaching the set destination is established at 20 to ensure the intelligent agent recognizes the importance of reaching the specific goals and to maximize motivation towards the target location.

(8) $$R_{\mathrm{arrive}}\left\{\begin{array}{cc} r=20\;\;\; & \mathrm{if}d_{t}<{\mathrm{goal}}_{\mathrm{dist}}\\ 0\;\;\; & \mathrm{else} \end{array}\right.$$

Obstacle Penalty: In the process of path planning, it is inevitable that the agent will encounter obstacles. To mitigate collisions and incentivize the agent to avoid obstacles, a substantial negative penalty of −20 is imposed upon collision with an obstacle. The significant penalty value effectively reduces collision incidents, thereby enhancing navigation safety.

(9) $$R_{\mathrm{collision}}\left\{\begin{array}{cc} r=-20\;\;\; & \mathrm{if}\;\min l_{i}\;<{\;\mathrm{collision}}_{\mathrm{dist}}\\ 0\;\;\; & \mathrm{else} \end{array}\right.$$

#### 3.3.2. Stepwise Guided Immediate Rewards

To further enhance navigation performance, an instant reward mechanism was developed to comprehensively assess the approach to the target, direction adjustment, and obstacle avoidance. This encourages the intelligent entity to efficiently and safely navigate towards the target point. When the mobile robot approaches the target point, it receives a positive reward if the current step is closer to the target than the previous step by more than 0; otherwise, it is penalized. This incentivizes progress towards the target. The angle difference (θ) between the robot’s current orientation and the target direction represents its movement accuracy; a larger difference necessitates greater adjustments and thus incurs a larger penalty value. This encourages improved movement towards the target. Additionally, it is essential to consider the distance between the mobile robot and obstacles during movement. When the robot moves too close to obstacles, a negative penalty is activated to prevent the robot from colliding. The design promotes safe path selection and mitigates potential losses and task failures resulting from collisions. The approach in this study enables the mobile robot to learn avoiding-obstacle strategies and selecting the safer paths, thereby enhancing overall navigation safety. The reword formula is as follows:

(10) R=d–l/2–abs(θ/π)

$d=d_{t}–d_{t-1}$ represents the difference in distance to the target between the current step and the previous step, and l is calculated as follows:

(11) l=1–min⁡li0   ifmin⁡ii<1else

where min $l_{i}$ represents the minimum distance to an obstacle within the radar detection range. If the value is greater than 1, no action is taken, indicating that the mobile robot is still relatively far from obstacles and within a safe environment—no rewards or penalties are applied. However, if the value is less than 1, it indicates that the mobile robot is too close to an obstacle and in a dangerous position. The difference between this value and 1 is calculated—the greater the difference, the closer the mobile robot is to the obstacle, and thus, the greater the penalty. After training, the mobile robot will be able to appropriately avoid obstacles.

By comprehensively considering three key factors of path optimization, orientation adjustment, and obstacle avoidance in the reward function, the approach guides the agent to efficiently and safely navigate complex environments towards the target.

### 3.4. Design of a Dual-Layer Noise Exploration Mechanism

When training the DDPG algorithm, incorporating noise can enhance the model’s exploration capabilities and optimize its decision- making strategy. This study implements a dynamically adjusted exploration noise strategy: action $a_{t}=\mu \left(s_{t}|\theta^{\mu }\right)+N_{t}$ is taken at each time step, with $N_{t}$ representing the noise during exploration. The noise is sampled from a normal distribution with a mean of 0 and a standard deviation of 1 in the initial iteration step to ensure extensive exploration of the action space and avoid falling into local optima. As the mobile robot progresses through each step, the value of exploration noise $N_{t}$ decreases by 0.00001, ultimately converging to a normal distribution with a mean of 0 and a standard deviation of 0.5. This enables the algorithm to prioritize fine-tuning established strategies in later stages, thereby enhancing convergence speed and performance. Furthermore, incorporating noise with a mean of 0 and a standard deviation of 0.5 into the target actions during sampling training can mitigate overfitting and bolster the robustness of estimated target Q-values. The dual-layer noise exploration mechanism not only amplifies the algorithm’s exploration capabilities but also optimizes its generalization ability and learning efficiency, ultimately leading to improved performance of the DDPG algorithm in complex continuous action spaces.

## 4. Simulation and Experiment

**Figure 2:**
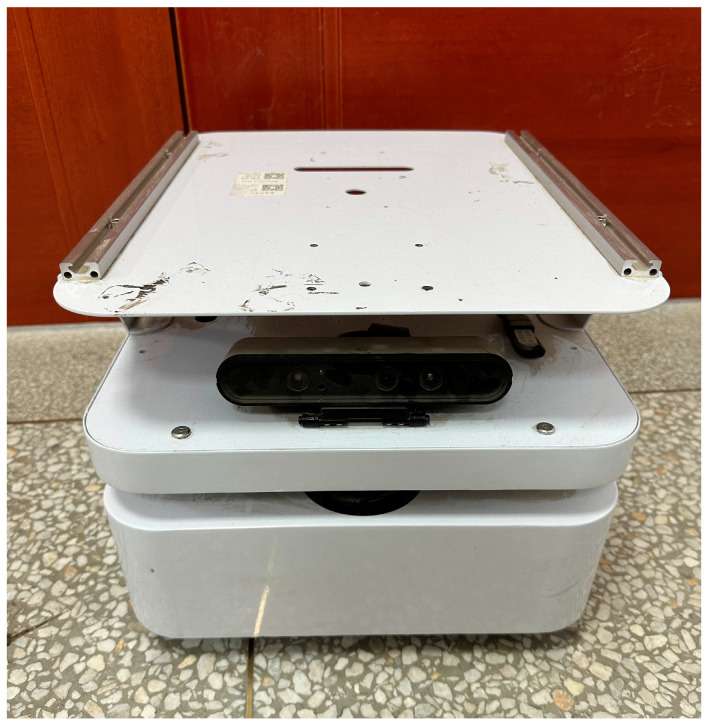
Mobile robot.

To simulate and verify the effectiveness of the improved DDPG algorithm in mobile robot path planning, a simulation experiment platform was built in this study based on ROS (Robot Operating System) and Gazebo. ROS offers robust functional support and broad hardware compatibility, while Gazebo serves as a high-fidelity 3D dynamics simulator that realistically replicates the behavior and interactions of robots in real environments. The Pioneer3dx two-wheeled differential drive robot was used as the simulation model in the platform due to its stable performance and extensive range of applications, which can accurately simulate the robot’s motion and path planning process in real-world settings. Through seamless integration of ROS and Gazebo during the simulation process, real-time monitoring and adjustment of the robot are facilitated to validate the effectiveness and robustness of the improved DDPG algorithm. The real-vehicle test trials were conducted using a mobile platform, as depicted in Figure 2. The platform utilizes a Jetson Nano as the control unit, which is a high-performance, low-power embedded computing device specifically designed for AI and robotics applications. The Jetson Nano offers robust computational capabilities and a comprehensive set of I/O interfaces, enabling it to effectively process robot motion control and sensor data. Additionally, the mobile platform is equipped with the RAMSON N10 LiDAR, which boasts high accuracy and a wide measurement range capable of capturing detailed real-time environmental information to provide the robot with precise environmental sensing capabilities. Through the integration of a high-level programming interface and underlying hardware control, precise movement control of the robot is achieved, allowing it to efficiently and safely execute path planning and navigation in complex environments. Through rigorous testing and validation in both simulated and real-world environments, the efficacy of the improved DDPG algorithm in mobile robot path planning can be accurately assessed to ensure its robustness and dependability across diverse environments. This dual validation approach not only facilitates a comprehensive performance evaluation, but also yields valuable insights and empirical evidence for further optimization and enhancement.

### 4.1. Simulation Environment Setup

**Figure 3:**
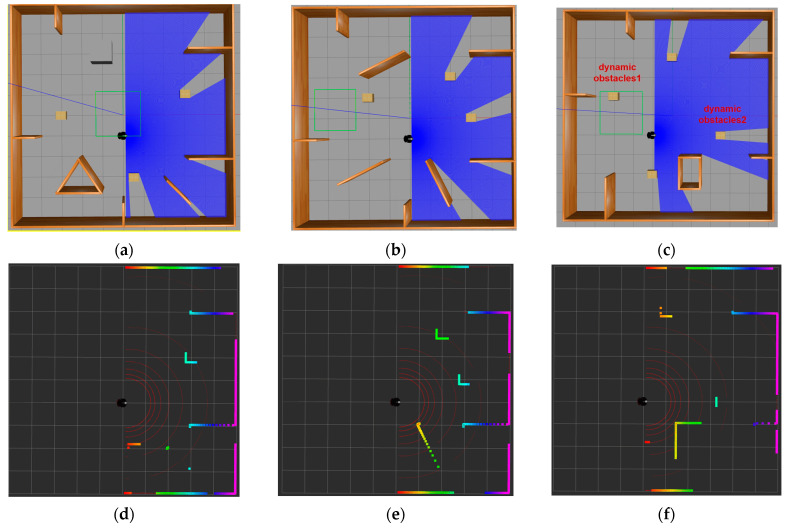
Simulation training environment (**a**) static simulation environment 1; (**b**) static simulation environment 2; (**c**) dynamic simulation environment; (**d**) visualization of static simulation environment 1 in Rviz; (**e**) visualization of static simulation environment 2 in Rviz; (**f**) visualization of the dynamic obstacle environment in Rviz.

In Gazebo, a 10 m × 10 m enclosed simulation environment was constructed using wooden boards. Various types of obstacles were then strategically placed within the environment to meet specific requirements. As depicted in Figure 3, the black object represents the Pioneer3dx robot, and the blue shaded area in the front 180° denotes the detection range of the LiDAR sensor. The initial position for the static simulation environment was defined at (0, −1), while the dynamic environment was set at (−1, 1). Target points were randomly positioned outside of obstacles within the environment to simulate the uncertainty and complexity encountered by the robot in real-world environments. Figure 3a illustrates the layout of static simulation environment 1, with all wooden-colored objects representing static obstacles. Figure 3b illustrates the layout of static simulation environment 2, with all wooden-colored and gray objects representing static obstacles. Figure 3d–f show static and dynamic simulation environments in Rviz, respectively. Rviz is a 3D visualization tool utilized within the ROS framework to visually represent the robot, sensor data, and environmental models. The depicted colored areas in these visualizations correspond to obstacles detected by the LiDAR, with different colors denoting distinct distances and positions of the obstacles, thereby providing comprehensive environmental information. Additionally, the red curves situated in front of the mobile robot are its detection range, effectively illustrating the scanning status of the LiDAR. Through the visualization tool, the navigation path, detection range, and obstacle avoidance behavior of the robot in the environment can be more clearly observed. Figure 3c is the configuration of the dynamic simulation environment, with two dynamic obstacles indicated alongside static obstacles. Dynamic obstacle 1 executes a back-and-forth linear motion along the *X*-axis within the interval (−3, 2), while dynamic obstacle 2 follows a similar pattern along the *Y*-axis within the interval (−4, 4). The motion equations governing these dynamic obstacles are as follows:

(12) $$\mathrm{box}1\_x=\left(T\;\mathrm{mod}\;\left(2\times \left(X_{\max}-X_{\min}\right)\right)\right)-\;(X_{\max}-X_{\min})$$

(13) $$\mathrm{box}2\_y=\left(T\;\mathrm{mod}\;\left(2\times \left(Y_{\max}-Y_{\min}\right)\right)\right)-\;(Y_{\max}-Y_{\min})$$

where T is the current time, $X_{\max}$ and $X_{\min}$ denote the maximum and minimum allowable movements along the *X*-axis, and $Y_{\max}$ and $Y_{\min}$ denote the maximum and minimum allowable movements along the *Y*-axis.

### 4.2. Analysis of Simulation Results

The mobile robot continuously explores and learns in the simulation environment. If it successfully reaches the target point or encounters an obstacle, or reaches the set maximum of 300 steps, the simulation is reset for the next round of training. Some parameters used during the simulation process are shown in Table 2:

#### 4.2.1. Static Obstacle Simulation

To compare the performance between the proposed improved DDPG algorithm and the traditional DDPG algorithm, two sets of simulation experiments comprising 1800 rounds each were designed: one in a relatively simple environment and the other in a more complex environment. The maximum number of action steps per round was set to 300 to ensure consistency in experiment conditions. The detailed discussion and analysis focus on four aspects: success rate, reward function curve trend, path length, and number of steps per round.

**Figure 4:**
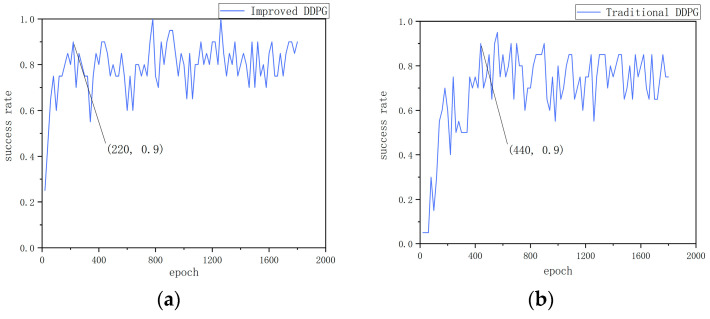

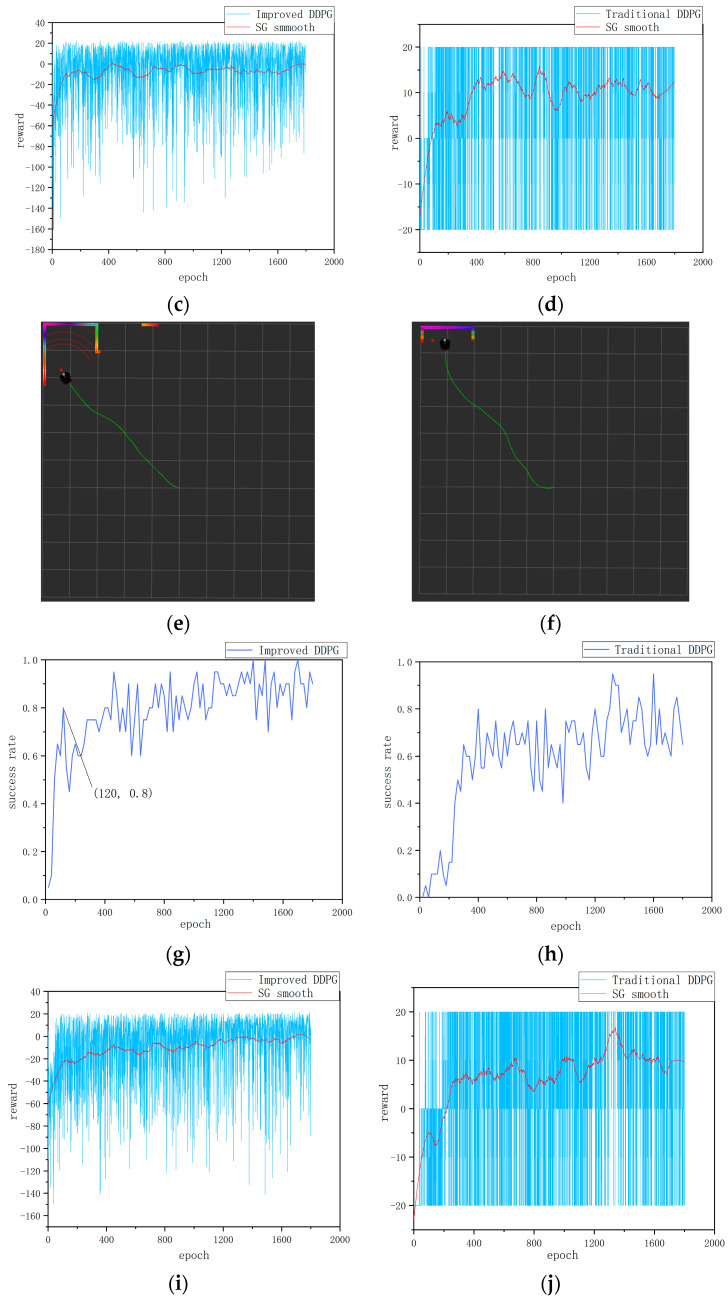

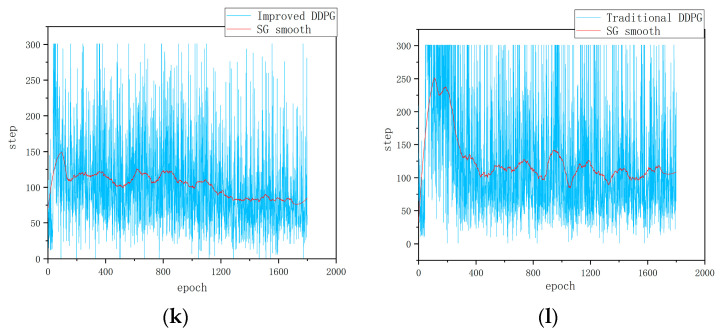
Static obstacle training results: (**a**) Success rate of the improved DDPG algorithm in environment 1. (**b**) Success rate of the traditional DDPG algorithm in environment 1. (**c**) Cumulative reward per round for the improved DDPG in environment 1. (**d**) Cumulative reward per round for the traditional DDPG in environment 1. (**e**) Path visualization in RViz for environment 1 with the improved DDPG algorithm. (**f**) Path visualization in RViz for environment 1 with the traditional DDPG algorithm. (**g**) Success rate of the improved DDPG algorithm in environment 2. (**h**) Success rate of the traditional DDPG algorithm in environment 2. (**i**) Cumulative reward per round for the improved DDPG algorithm in environment 2. (**j**) Cumulative reward per round for the traditional DDPG algorithm in environment 2. (**k**) Cumulative steps per round for the improved DDPG algorithm in environment 2. (**l**) Cumulative steps per round for the traditional DDPG algorithm in environment 2.

Figure 4a–f show the experimental data for environment 1. As can be seen from Figure 4a,b the improved algorithm reaches a 90% success rate at the 220th round and stabilizes at around 85% after the 1100th round. In contrast, the traditional algorithm only reaches a 90% success rate at the 440th round, and the success rate stabilizes at around 65% after the 1600th round. This indicates that the multi-step update mechanism in the improved algorithm plays a key role, not only accelerating the convergence speed but also improving convergence stability. From Figure 4c,d it can be seen that the improved algorithm experiences significant fluctuations initially, indicating that the mobile robot engages in extensive exploration in the early stages. However, it starts to stabilize after the 1000th round, whereas the traditional algorithm remains in a state of oscillation throughout. This suggests that the dual-noise design in the improved algorithm is reasonable, increasing the exploration rate of the mobile robot. At the same time, it also demonstrates that the improved reward function effectively guides the mobile robot’s actions. From Figure 4e,f it can be seen that the improved algorithm has a higher level of real-time performance, as the mobile robot can quickly adjust its orientation towards the target point from the outset, resulting in a smoother path. This further underscores the importance of an appropriately designed reward function.

Figure 4g–l show the experimental data in environment 2. First, as shown in Figure 4g,h the path planning success rate trends in a static obstacle environment for two algorithms are illustrated. From Figure 4g,h it can be seen that the success rate of the traditional DDPG algorithm reaches 80% at the 400th round, after which it fluctuates significantly, hovering between 40% and 80%, and finally stabilizes at around 65%, indicating its uncertainty and adaptability limitations in path planning. In contrast, the improved DDPG algorithm reaches an 80% success rate at the 120th round and maintains around 85% throughout the training cycle, with a maximum success rate reaching 100%. Compared with the traditional DDPG algorithm, the stability of the success rate of the improved algorithm increases by 20%; the results demonstrate improvements on the DDPG’s learning efficiency and strategy robustness. Second, as shown in Figure 4i,j the cumulative reward value per round further reflects the algorithm’s performance. The improved DDPG algorithm maintains an overall high and stable level of cumulative rewards with less volatility, demonstrating more stable path planning and obstacle avoidance performance. In contrast, the cumulative reward of the traditional DDPG algorithm is more volatile, oscillating between 20 and −20. This large volatility reflects the instability of the traditional algorithm in complex environments and the importance of reward function design for path planning and obstacle avoidance. Finally, as shown in Figure 4k,l the cumulative number of steps per round further verifies the efficiency of the improved algorithm. It can be seen that the number of steps with the improved algorithm per round gradually decreases, ultimately leading to a more efficient path planning strategy. Compared with the traditional algorithm, the number of steps taken by the improved algorithm to reach the target point is reduced by 25%, indicating that the improved algorithm continuously optimizes path selection during the iteration process, improving the efficiency and accuracy of path planning.

The above comparative analyses demonstrate that the improved DDPG algorithm outperforms the traditional DDPG algorithm in several performance metrics. First, in terms of learning efficiency and strategy robustness, the improved DDPG algorithm achieves a success rate of 80% in a relatively short 120-round training time and maintains a high success rate and low volatility in the subsequent training process, showing high learning efficiency and robustness. Second, on the part of accumulated reward value, the improved DDPG algorithm maintains high stability, reflecting its stable performance in path planning, while the traditional algorithm has large fluctuations in reward value, showing instability when dealing with complex environments. Finally, in the path planning efficiency, the improved algorithm shows a gradual reduction in the number of steps required in each round, and ultimately reduces the number of steps by 25% compared with the traditional algorithm, indicating that the improved algorithm continuously optimizes the path selection during iteration and improves the efficiency of path planning.

#### 4.2.2. Dynamic Obstacle Simulation:

**Figure 5:**
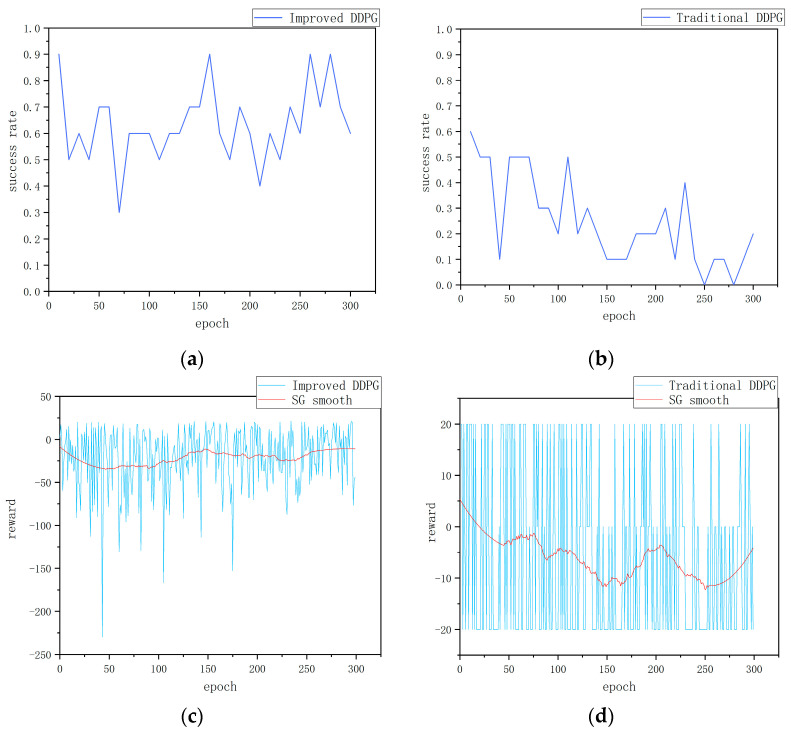
Dynamic obstacle training results: (**a**) success rate of the improved DDPG algorithm, (**b**) success rate of the traditional DDPG algorithm, (**c**) cumulative reward per round for the improved DDPG algorithm, and (**d**) cumulative reward per round for the traditional DDPG algorithm.

The improved DDPG algorithm model and the traditional DDPG algorithm model, both initially trained in a static environment, are subsequently transferred to a dynamic obstacle simulation environment for further training over 300 episodes. The outcomes of this training are depicted in Figure 5. The performance of the algorithm is evaluated from two aspects: the success rate and the value of the per-episode reward function.

Firstly, from the perspective of success rate, Figure 5a,b show the change in success rate of the two algorithms in the dynamic obstacle environment. The improved DDPG algorithm shows a high success rate during training, reaching 90% or more several times and stabilizing at around 65%. This indicates that the improved algorithm is able to effectively adapt and navigate in the dynamic obstacle environment and exhibits strong generalization ability and robustness. In contrast, the traditional DDPG algorithm’s success rate decreases gradually during training, dropping to 0 at around 250 and rising to 20% at around 300. This significant difference indicates that the traditional algorithm has poor adaptability in the face of dynamic obstacles, while the improved algorithm can quickly adapt to environmental changes and maintain a high success rate.

Secondly, with regard to the reward function value per round, Figure 5c,d show the changes in the cumulative reward value per round of the two algorithms during training. Figure 5c shows the change in the reward value of the improved DDPG algorithm. Although there are some fluctuations in the cumulative reward value per round, the smooth curve gradually stabilizes, indicating that the improved algorithm is able to continuously accumulate rewards in a dynamic environment and gradually form an effective navigation strategy. This further verifies the stability and effectiveness of the improved algorithm. In contrast, Figure 5d shows the change in the reward value of the traditional DDPG algorithm. Even after smoothing, the cumulative reward curve still shows significant fluctuations, indicating that traditional method has difficulty forming stable and effective navigation strategies with weak cumulative reward capabilities. This fluctuation reflects the shortcomings of traditional methods in dealing with dynamic environments, as they fail to effectively adjust themselves to continuously optimize their strategies.

In conclusion, through the comparative analysis of success rate and per-episode reward function value, it is clear that the improved DDPG algorithm shows significant advantages in a dynamic obstacle environment. The improved DDPG algorithm surpasses the traditional algorithm not only in terms of success rate but also in accumulated reward stability. These results fully indicate that the improved DDPG algorithm has stronger adaptability and robustness, and is capable of effectively navigating in complex dynamic mapless environments.

### 4.3. Real Mobile Robot Experiment

In order to ascertain the viability of the proposed algorithm in a real-world setting, two sets of mobile robot test experiments were conducted in this study. The results of each set of experiments were subsequently analyzed to evaluate the performance of the algorithm. The evaluation metrics include the movement trajectory of the mobile robot and the extent to which it successfully reaches the target point. The improved DDPG algorithm model, obtained after simulation training, was embedded into the real robot system during the test period. The mobile robot program was initiated after the target point was set, whereby the mobile robot obtained feedback data from the radar sensors, its precise coordinates, and instantaneous velocity information by subscribing to the relevant topics in real time. Subsequently, the data were processed by a deep neural network and converted into standard input states consistent with the simulation environment. The policy network computed the linear and angular velocities, which were subsequently delivered to the mobile robot through specific topics to update the state in real time. The data were then processed by a deep neural network and transformed into a standardized input state, which was required by the strategy network. This network calculated the linear and angular velocities which were used to guide the mobile robot towards the target point.

**Figure 6:**
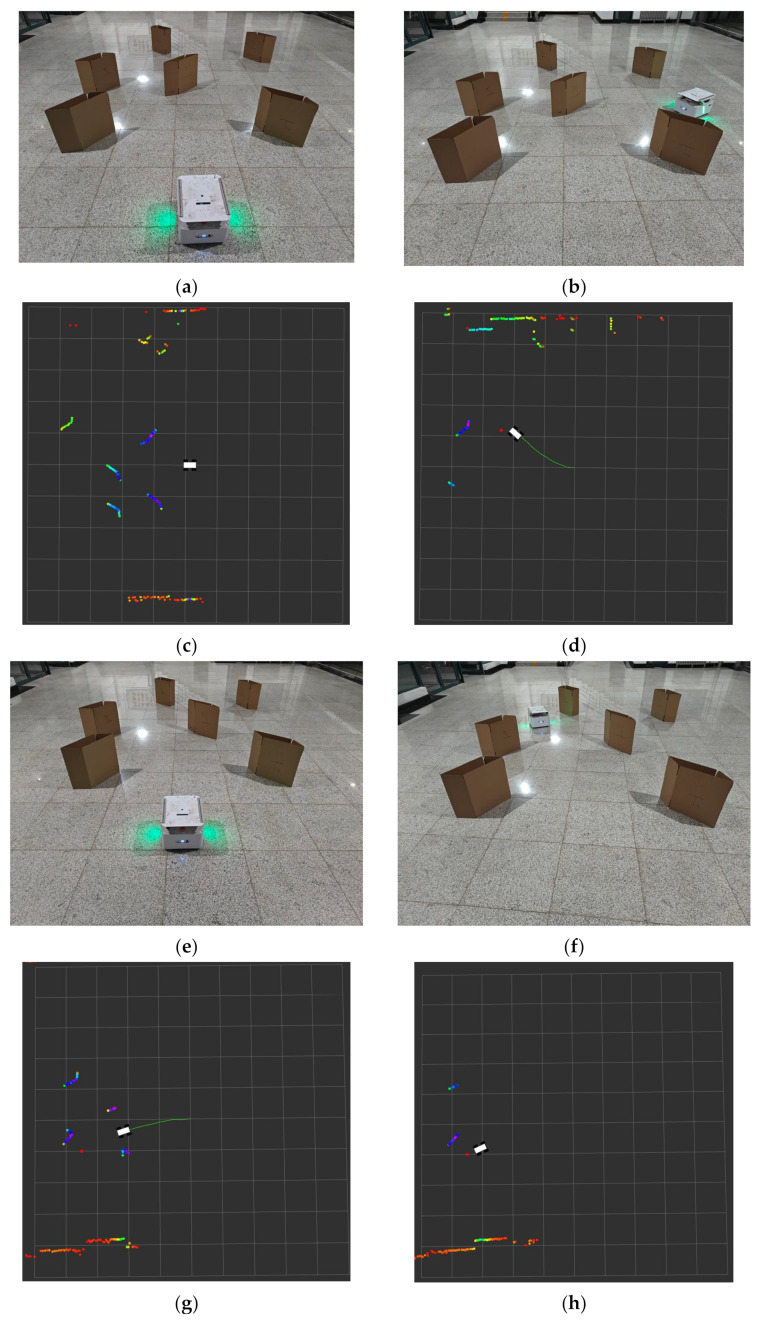
Real mobile robot test experiment results: (**a**) mobile robot initial scene one, (**b**) mobile robot endpoint scene one, (**c**) Rviz visualization of mobile robot initial scene one, (**d**) Rviz visualization of mobile robot endpoint scene one, (**e**) mobile robot initial scene two, (**f**) mobile robot endpoint scene two, (**g**) Rviz visualization of mobile robot progress, and (**h**) Rviz visualization of mobile robot endpoint scene two.

Figure 6a,b show the initial position of the mobile robot and the location of the target point it reaches, and Figure 6c,d demonstrate the visualization results via the Rviz tool. The colored portions shown as Figure 6a, b depict the obstacles identified by the radar on the robot; the small red dots represent the location of the target point, and the green line illustrates the cart’s trajectory, which, as evidenced in Figure 6d, successfully reached the target point. Furthermore, the robot trajectory is notably smooth, which indicates that the cart is capable of effectively recognizing obstacles and planning an optimal path to the target point. The second set of experiments provides further confirmation of the efficacy of the algorithm. Figure 6e,f also illustrate the initial position of the robot and the designated goal point. The Rviz visualization results in Figure 6g,h demonstrate the smoothness and directness of the robot’s trajectory, indicating that the robot selects the most direct path in approaching the goal point without detours.

The combination of the results from the two sets of experiments demonstrates that the improved DDPG algorithm not only performs well in a simulated environment but also exhibits robust performance and accuracy in a physical robot system. The robot is capable of rapid adaptation to environmental changes, precise obstacle recognition, and the effective planning of an optimal path. It validates the feasibility and efficacy of the enhanced algorithm in practical applications and provides substantial technical support for the future autonomous navigation of mobile robots in complex environments.

## 5. Conclusions

In this study, the DDPG algorithm is optimized to improve the path planning capability of mobile robots in mapless environments. By using an optimized state and action space, introducing a multi-stage update strategy, improving the reward function, and adopting a two-layer noise mechanism, the learning efficiency and navigation performance of the algorithm are significantly improved, and its adaptability and robustness in complex environments are enhanced. First, the redesigned state and action spaces are more in accordance with the actual needs of mobile robots in mapless environments. The experimental results show that the improved algorithm has a navigation success rate of up to 100% in static obstacle environments and stabilizes at about 85%, with a 20% increase in stability compared to the original algorithm. In addition, the pathfinding path is smoother and the number of steps required is reduced by 25%, indicating that the optimized state and action space design can effectively improve the efficiency and accuracy of path planning. Second, the introduced multi-step update strategy and improved reward function enable the algorithm to better accumulate long-term rewards, thus improving the robustness of path planning. In the dynamic obstacle environment, the success rate of the improved algorithm is increased from the original 20% to 65%, demonstrating that the optimized reward mechanism and multi-step update strategy can effectively improve the adaptability and stability of the algorithm in a complex environment. Finally, the adopted two-layer noise mechanism makes the algorithm more flexible in the exploration process, avoids falling into local optimal solutions, and improves the overall navigation performance. The feasibility and effectiveness of the algorithm in real environments are further verified through real mobile robot tests. The experimental results show that the mobile robot can quickly adapt to environmental changes, accurately identify obstacles, and effectively plan the optimal path, demonstrating the robustness and generalization ability of the improved algorithm in different environments. In conclusion, the improved DDPG algorithm performs well in complex path planning tasks, which not only improves the navigation success rate and path planning efficiency in static and dynamic obstacle environments, but also demonstrates its strong adaptive ability and reliability in real applications through real mobile robot tests, providing an effective solution for autonomous navigation of mobile robots in mapless environments.

## Figures and Tables

**Table 1:** Pseudocode for interaction and storage.

Interaction and Storage
$\mathrm{Initialize}\;\mathrm{policy}\;\mathrm{network}\;\mathrm{parameters}\;\theta^{\mu }$ $\;\mathrm{and}\;\mathrm{value}\;\mathrm{network}\;\mathrm{parameters}\;\theta^{Q}$
For each time step t:
Based on current state $S_{t}$ using the policy network to generate action
$a_{t}=\mu \left(S_{t}|\theta^{\mu }\right)+N_{t}$
$\mathrm{Execute}\;\mathrm{action}\;a_{t}$ $,\;\mathrm{obtain}\;\mathrm{new}\;\mathrm{state}\;S_{t+1}$ $\;\mathrm{and}\;\mathrm{reward}\;r_{t}$
If Task Completed or FailureState: done = True
Else: done = False
$\mathrm{Store}\;\mathrm{the}\;\mathrm{experience}\;(S_{t},a_{t},r_{t},S_{t+1},\mathrm{done})$ into replay buffer
End for

**Table 2:** Training parameter settings.

Parameter	Value
Actor Network Learning Network	0.0001
Critic Network Learning Rate	0.0001
Target Network Soft Update Parameter	0.005
Discount Factor	0.99
Experience Pool Size	100,000
Batch Size	64
Collision Threshold	0.35 m
Distance to Target Threshold	0.4 m
Maximum Linear Speed	1 m/s
Maximum Angular Speed	1 rad/s

## Data Availability

The data presented in this study are available on request from the corresponding author.
